# The diagnosis of acute interstitial nephritis caused by infection versus antibiotic-induced interstitial nephritis: a narrative review

**DOI:** 10.1093/ckj/sfae054

**Published:** 2024-03-04

**Authors:** Amir Muhammad, Yingli Zhang, Ling Huang, Qiongjing Yuan, Wei Wang, Jiaxi Pu, Wei Lin, Rong Tang, Xiangcheng Xiao

**Affiliations:** Department of Nephrology, Xiangya Hospital, Central South University, Changsha, China; Department of Nephrology, Third Hospital of Changsha, Changsha, China; Department of Nephrology, Xiangya Hospital, Central South University, Changsha, China; Department of Nephrology, Xiangya Hospital, Central South University, Changsha, China; Department of Nephrology, Xiangya Hospital, Central South University, Changsha, China; Department of Nephrology, Xiangya Hospital, Central South University, Changsha, China; Department of Pathology, Xiangya Hospital, Central South University, Changsha, China; Department of Nephrology, Xiangya Hospital, Central South University, Changsha, China; Department of Nephrology, Xiangya Hospital, Central South University, Changsha, China

**Keywords:** acute interstitial nephritis, antibiotics, biomarkers, chronic kidney disease, infection

## Abstract

Acute interstitial nephritis (AIN) is a significant contributor to acute kidney injury and can be attributed to a variety of factors, including but not limited to allergens or drugs, infections, autoimmune or systemic diseases, and idiopathic forms of the disease. In some cases, AIN requires a therapeutic action according to a single specific etiology by handling the offending agent and applying an immunosuppressant. Although AIN can be diagnosed through renal biopsy, it is not able to pinpoint the precise cause when multiple causes are suspected to be present simultaneously. Such situations arise when a patient suffering from infection develops AIN during antibiotic therapy, the exact causative factor of which becomes a challenge for the clinicians to determine. This is attributed to the different approaches employed in different etiologies, wherein clinicians are required to maintain the current antibiotic therapy or augment the dose in cases of infection as AIN etiology, without resorting to immunosuppressant therapy as the primary objective is infection killing. In contrast, antibiotics as an etiology for AIN require an alternative drug from the antibiotics group, along with an immunosuppressant. In the interim, delaying the identification of the precise cause may result in interstitial fibrosis and chronic kidney disease. This narrative review highlights certain findings that can be typical of infection-associated ATIN compared with antibiotic-associated ATIN based on clinical history and physical examination, clinical presentation of different antibiotic drug classes, histopathological features, classical and novel biomarkers, serum and urine cytokines and chemokines, cellular biomarkers, and genetic biomarkers. Although these findings cannot provide conclusive and clear recommendations that can be useful in the clinical practice, they can entice researchers to conduct original research on these features to discover clear recommendations.

## INTRODUCTION

**Figure 1:**
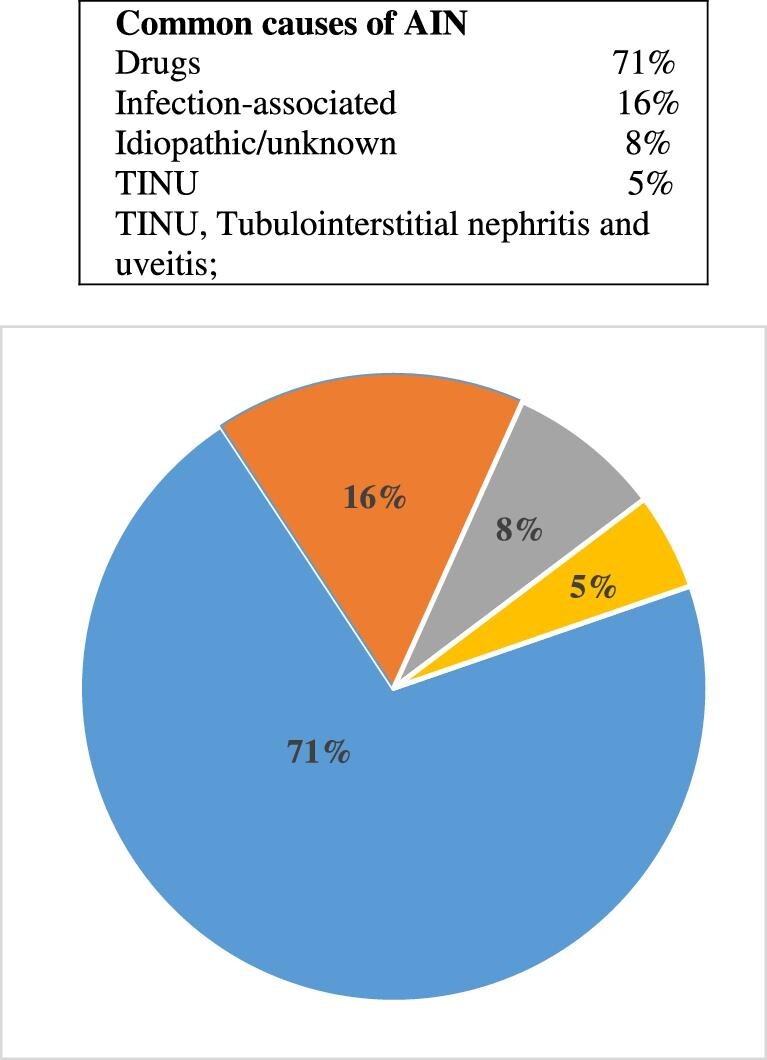
Common causes of AIN. This figure is based on data from Baker and Pusey [11].

**Figure 2:**
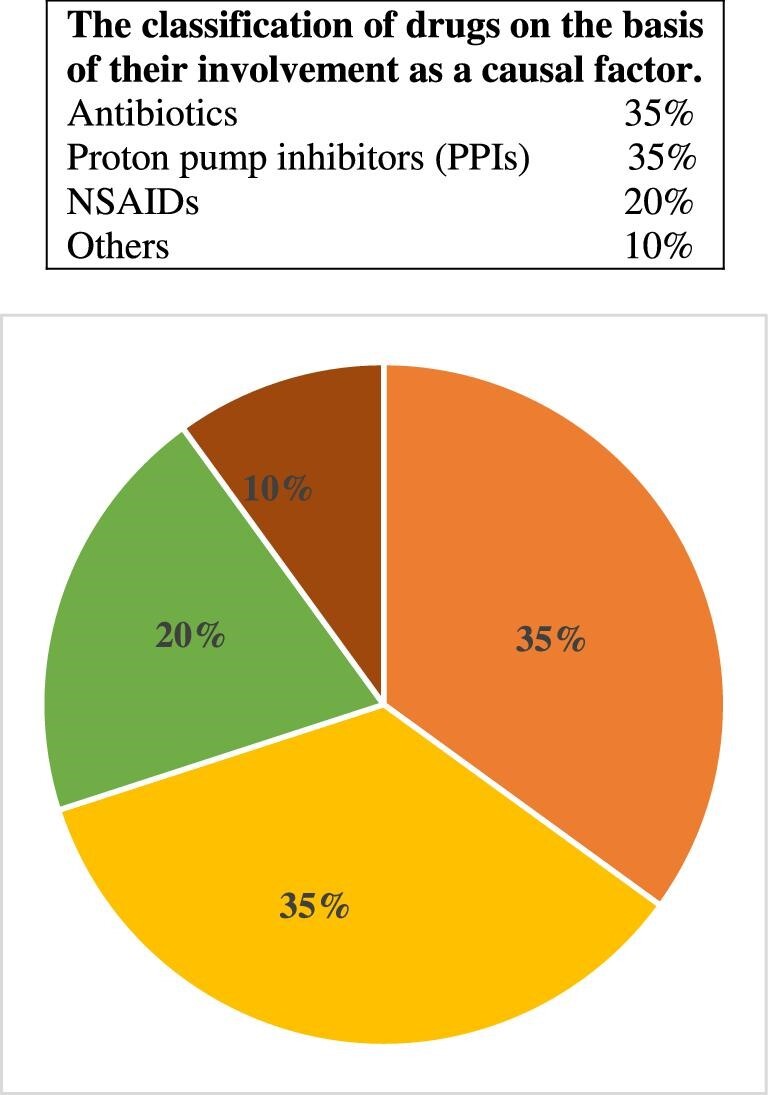
Based on data from Valluri *et al*. [12].

Acute tubulointerstitial nephritis (ATIN), or AIN, is an immunomediated disease that affects the tubulointerstitial area of the kidneys, accompanied by histological findings of interstitial inflammation, edema and tubulitis [1]. The tubulointerstitial area comprises 80% of the renal surface area and is composed of cellular and extracellular matrix components [2]. AIN is an important cause of acute kidney injury (AKI), with various etiologies such as allergens or drugs, infections, autoimmune or systemic diseases, and idiopathic forms of the disease [3]. It is estimated that up to 10%–27% of hospitalized AKI patients are affected by AIN, making it the third most prevalent cause of hospital-acquired AKI after acute tubular necrosis (ATN) and prerenal AKI [4–9]. In developed countries, the most common cause of AIN is an immunoallergic drug reaction, accounting for 70%–90% of cases [7, 10]. In a 2004 report based on pooled data from three large studies, antibiotics were accounted for one-third of the cases from the drug-induced etiology in 91 of the 128 cases (71.1%) [11] (Fig. 1). Among the remaining 37 cases, 20 were induced by infection, 10 were idiopathic, 6 had tubulointerstitial nephritis with uveitis (TINU) and 1 had sarcoidosis [11]. A study conducted by Valluri *et al*. [12] (Fig. 2) in 2015, based on 171 biopsy-confirmed cases between 2000 and 2012, revealed that antibiotics accounted for 35% of the cases, proton pump inhibitors (35%), and non-steroidal anti-inflammatory drugs (NSAIDs) (20%) were identified as the primary culprits. Therefore, a significant number of AIN cases can be caused by antibiotics and infections (Figs 1 and 2).

The renal biopsy procedure is widely recognized as the most reliable diagnostic method for AIN, however, it is not effective in identifying the underlying cause. Serious complications arise when a patient who is infected develops AIN during antibiotic therapy. This is due to the fact that both infection and antibiotics can be suspected as the offending agent simultaneously, resulting in misguided efforts to identify the offending agent, as both etiologies may exhibit similar histopathological or clinical characteristics. Furthermore, the two etiologies require different therapeutic actions because infection as AIN etiology requires the continuation of current antibiotic therapy or even an increase in dose without the need for immunosuppressant therapy [13–15]. Treating the underlying infection and supportive therapy is the main initial target. On the other hand, during the case of an antibiotic-related AIN, clinicians may require the use of alternative antibiotic drugs along with the application of an immunosuppressant [3, 6, 16, 17]. The delay in finding the exact cause can lead to interstitial fibrosis and chronic kidney disease (CKD) [1, 3, 11, 13, 18, 19], and research estimates that 40% to 60% of patients develop CKD after an episode of AIN [20–25].

Clinicians are required to conduct prompt diagnosis, identification and withdrawal of the offending agent, as these are crucial factors in preserving kidney function and ensuring a favorable long-term renal prognosis [26]. A recent case series published by Fernandez-Juarez *et al*. [27] demonstrated that the culprit drug cannot be precisely identified in nearly 30% of cases. This lack of crucial information hinders the primary therapeutic option, as it entails the withdrawal of the causative agent. Furthermore, it may increase the probability of developing recurrences and partial renal recovery [1, 11, 18]. Barreto *et al*. [13] demonstrated that the initial inflammatory lesions in AIN can commence to progress into irreversible interstitial fibrosis as early as 7 days after drug exposure. This may be the reason why, despite the most effective treatment options, only 40%–50% of patients with AIN experience complete recovery of kidney function [28]. AIN may also represent an earlier clinical manifestation of an underlying systemic or infectious disease with fewer extrarenal manifestations [29–31], thereby hindering the accurate diagnosis and treatment [5].

Drugs, particularly antibiotics, are known to cause AIN, which is a drug hypersensitivity reaction (DHR) that manifests itself within 7–10 days of exposure to the offending drug [20] (Tables 1 and 2 [32–49]). However, this time can be shorter if the patient is exposing to the same drug for the second time [25], thus misguiding in finding the culprit drug. AIN can also arise as a reactive, cytokine-mediated response to infection, as was first described in 1898 by Councilman for streptococcal infections [50] (Table 1 [38–48]). The exact pathomechanism of infection-induced AIN is not yet clear, though some mechanisms have been proposed. Certain microorganisms that act as planted antigens have the ability to accumulate in the interstitium, mimic a normally present antigen in the tubular basement membrane and elicit an immune response directed against this antigen (Table 1) [32–48]. Furthermore, the direct cytopathic effects of microbial antigens or cytokine-mediated inflammation may be the explanation for the renal injury [14, 51] (Table 1 [32–48]). In order to gain a more thorough comprehension of the pathophysiology and etiology associated with early diagnosis, researchers are endeavoring to identify reliable biomarkers of the disease, with a particular emphasis on urinary cytokines and chemokines that may manifest renal local inflammation (Table 3) [52–62]. There is a growing interest in finding cell-based tests that can identify the exact drug involved in hypersensitivity reactions to drugs, manifesting as AIN or ATIN [63]. Some studies also found that certain single-nucleotide polymorphisms in human leukocyte antigens (HLA) or cytokine genes confer susceptibility to some etiologies inducing ATIN [64]. Hence, it is imperative for researchers to conduct additional research on these procedures to facilitate clinicians in the timely identification of the primary aggravating factor causing ATIN, thereby aiding in diagnosis, prognosis and follow-up.

**Table 1:** Literature review of antibiotics-induced AIN and infection-induced AIN.

Offending agent	Associated clinical features	Diagnostic procedures	Pathomechanism or immunogenecity	Biopsy findings	Therapy	Outcome
Antibiotics						
BLs	Fever and rash, eosinophilia, eosinophiluria and pyuria	Renal biopsy, physical examination clinical features and history	Delayed T-cell-mediated hypersensitivity reaction	Interstitial infiltration with mononuclear cells	Discontinuation of culprit drug and administration of steroid	Higher chances of recovery
Sulfonamides	Fever and rash, eosinophilia, eosinophiluria and pyuria	Renal biopsy, physical examination clinical features and history	Delayed T-cell-mediated hypersensitivity reaction	Interstitial infiltration with mononuclear cells	Discontinuation of sulfonamides and administration of steroid	Higher chances of recovery
Fluoroquinolones (Hung *et al*., *Nephrol Dial Transplant* 2006) [32]	Eosinophilia and pyuria, fever, elevated WBC count and anuria	Renal biopsy, physical examination clinical features and history	Delayed T-cell-mediated hypersensitivity reaction	Mild hypercellularity, interstitial infiltration with mononuclear cells	Discontinuation of fluoroquinolones	Recovered
Rifampicin (Salih *et al*., *Saudi J Kidney Dis Transpl* 2008) [33]	Fever and rash, eosinophilia, eosinophiluria, pyuria, abdominal pain, high leukocyte count, decrease in urine pH	Renal biopsy, physical examination and clinical history	Rifampicin-dependent antibodies, especially IgM	Acute tubular necrosis with mild tubulo-interstitial mononuclear cellular infiltrate	Rifampicin was discontinued, without steroid	Recovered
Vancomycin (Kannan *et al*., *Front Med* 2022) [34]	Inconsistent pyuria, generalized fatigue, fever, elevated WBC count, moderate hematuria	Blood culture positive for gram positive cocci, vancomycin trough was 17 µg/mL	Delayed T-cell-mediated hypersensitivity reaction	Diffuse cellular infiltrate within the interstitium with inflammatory cells including eosinophils and lymphocytes, numerous eosinophils in the interstitium	Administration of prednisone and discontinuation of vancomycin	Recovered
Minocycline (Sharma *et al*., *SAGE Open Med Case Rep* 2020) [35]	Generalized rash, anasarca, fever, myalgia, nausea, vomiting, sore throat and generalized body weakness, mild proteinuria	Renal biopsy, physical examination, clinical features and history	Delayed T-cell-mediated hypersensitivity reaction	Marked interstitial edema with patchy interstitial lymphoplasmacytic infiltrates	Corticosteroids, diphenhydramine, and discontinuation of minocycline	Recovered
Prothionamide (Asnake *et al*., *SAGE Open Med Case Rep* 2022) [36]	Fatigability, rash and intermittent fever, proteinuria, hematuria	Physical examination, clinical features and history	Delayed T-cell-mediated hypersensitivity reaction	NA	Discontinuation of prothionamide	Recovered
Infections						
Staphylococcus infection [(Raina *et al*., *J Nephropathol* 2017) [37]	Nausea, vomiting, poor oral intake, and decreased urination, elevated white blood cell count with predominant neutrophils, proteinuria, sterile pyuria	Urine culture, renal biopsy, physical examination, clinical features and history	Low C3, normal C4, and positive ANA (1:80 titer)	Patchy interstitial inflammatory infiltrates with prominent eosinophilic component	CRRT, vancomycin , piperacillin + tazobactam, clindamycin, prednisone after 7th day of hospitalization	Recovered
Salmonella infection (Caers *et al*., *Eur J Intern Med* 2006) [38]	Fever, malaise, vomiting, diarrhea, microscopic haematuria, pyuria, subnephrotic proteinuria, and normal or mildly reduced GFR	Renal biopsy, physical examination, clinical features and history	Immunofluorescence showed the absence of immunoglobulin deposits	Interstitial edema and lymphocytic infiltrate with plasma cells and rare granulocytes	Ciprofloxacin, ceftriaxone, methylprednisolone	Recovered
Legionella infection (Daumas *et al*., *J Med Case Rep* 2012) [39]	Aseptic leukocyturia, hematuria, proteinuria, anuric	*Legionella* antigenuria was positive, renal biopsy, physical examination, clinical features and history	NA	Interstitial cell infiltrate associated with edema and few tubules lined by flattened cells, focal tubulitis with mononuclear cells that have invaded few tubules	Erythromycin and ofloxacin, HD, steroid	Recovered
*Yersinia* infection (Iijima *et al*., *Am J Nephrol* 1989) [40]	Proteinuria, granular casts of urine, fever,	Renal biopsy, physical examination, clinical features and history	NA	Extensive lymphocyte infiltration and diffuse edematous change in the interstitium	Cephalexin, acetylsalicylic acid	CKD
*Brucella* infection (Dagli *et al*., *J Infect Dev Ctries* 2011) [41]	Weakness, generalized joint pain, sweats, nausea, loss of appetite and fever, dysuria, hematuria, proteinuria, granular casts	Urine culture, physical examination, clinical features and history	Brucella agglutinins were reported at a titer of 1:160, brucella indirect hemagglutination (Rose-Bengal) test was positive, antigen-antibody reaction as the cause of the renal damage	NA	Rifampin and doxycycline	Recovered
*Campylobacter jejuni* (Rautelin *et al*., *Scand J Urol Nephrol* 1987) [42]	Fever and backache, oliguric, hematuria, proteinuria, febrile	Isolation of *Campylobacter jejuni* heat-stable serotype 2 from faeces, renal biopsy, physical examination, clinical features and history	Elevated titer of *Campylobacter* IgA class antibody was detected, antigen-antibody reaction as the cause of the renal damage	Extensive interstitial inflammation, with mononuclear cells and fewer granulocytes; infiltrates in medullar and cortical areas	NA	Recovered after 22 days from the onset of symptoms
*Corynebacterium diphtheriae*	Sore throat, weakness, fever, and swollen glands	Blood or urine culture, physical examination, clinical features and history	NA	NA	A vaccine, DTaP, effectively prevents the disease, penicillin or erythromycin	NA
Streptococcal infection (Chang *et al*., *Nephrol Dial Transplant* 2011) [43]	Sore throat, diarrhoea and general myalgia, erythematous rash, proteinuria	Blood and urine culture grew Group A Streptococcus pyogenes, renal biopsy, physical examination, clinical features and history	Immunohistochemical study depicted diffusely strong positive signals of anti-streptococcal pyrogenic exotoxin B antibodies in tubular epithelial cells and tubulointerstitial compartments of the cortical tissue; antigen-antibody reaction as the cause of the renal damage	Inflammatory cells infiltration and edema in the interstitium of cortex, and was infiltrated by admixed inflammatory cells, including neutrophils, lymphocytes, plasma cells and especially oeosinophils	Four sessions of haemodialysis, supportive therapy, ceftriaxone	After 23 days of hospitalization, he recovered
*Streptococcus pneumoniae* (Phillips *et al*., *Pediatr Nephrol* 2005) [44]	Fever, cough, vomiting, abdominal pain and lethargy, increase in WBCs count	Blood and urine cultures grew penicillin-sensitive *S. pneumoniae*, physical examination, clinical features and history	NA	NA	Intravenous ceftriaxone and maintenance hydration	Recovered post 8 weeks of hospitalization
*Escherichia coli* (Kwon *et al*., *J Korean Med Sci* 2015) [45]	Hematuria, pyuria, oliguric acute kidney injury, acute pyelonephritis	Bacteremia and megalocytic interstitial nephritis, renal biopsy, physical examination, clinical features and history	NA	Infiltration of numerous histiocytes without Michaelis-Gutmann bodies, CD68 positivity in infiltrated histiocytes, mesangial staining was positive for C1q and electron microscopy showed moderate effacement of epithelial foot processes	CRRT, HD, cefotaxime (third-generation cephalosporin) and azithromycin, high-dose steroid	Renal function was not recovered despite adequate duration of susceptible antibiotic treatment, accompanied by negative conversion of bacteremia and bacteriuria
Melioidosis (Prabhu *et al*., *J Nephrol*. 2021) [46]	Hyponatremia, microscopic hematuria and proteinuria, renal and lower renal tract abscesses, pneumonia, abscess formation, sepsis, and multi-organ dysfunction	Positive culture for *Burkholderia pseudomallei*, bacteraemia, renal biopsy, physical examination, clinical features and history	NA	Acute tubular injury, interstitial nephritis and microabscesses	HD, ceftazidime and carbapenem with or without cotrimoxazole, or chlorampheniol, doxycycline and cotrimoxazole	AKI was predicted by bacteraemia and CKD, and was associated with higher mortality and need for ICU care; but renal function recovery was observed in survivors
Scrub *typhus* (Kim *et al*., *Kidney Res Clin Pract* 2013) [47]	Proteinuria, fever, chills, myalgia, skin rash, lymphadenopathy and an eschar	Renal biopsy, physical examination, clinical features and history	Immunochromatographic antibody assay test for *Orientia tsutsugamushi* was positive, and the immunofluorescent antibody assay test profile showed an IgM titer 41:2048 and IgG titer 41:2048	Diffuse lymphoplasmacytic infiltrations with scattered neutrophils in the edematous interstitium, tubulitis and acute tubular necrosis, detached tubular epithelial cells and neutrophils were detected in the tubular lumen, mild tubular atrophy	HD supportive therapy, doxycycline, azithromycin	Renal function did not improve 18 months after discharge and the patient required continuous HD; this failure to recover might be from cross reaction or superinfection from one fever type to other
*Leptospira* infection (Daher Ede *et al*., *J Bras Nefrol* 2010) [48]	Nonoliguric and hypokalemic, fever, chills, severe headache, followed by anorexia, diarrhea, nauseas, vomiting, malaise, myalgia, mild proteinuria and urinary sediment abnormalities	Blood culture, clinical findings and epidemiological data	IgM antibodies, MAT, results are considered positive when antibody titers are four times greater than the reference value	NA	Early and daily HD; low volume infusion and lung-protective strategies	Mortality in leptospirosis-associated AKI is around 22%

NA, not available; IgM, immunoglobulin M; CRRT, continous renal replacement therapy; C3, complement 3; C4, complement 4; ANA, antinuclear antibody; GFR, glomerular filtration rate; HD, hemodialysis; IgA, immunoglobulin A; DTaP, diphtheria, tetanus and acellular pertussis; CD68, cluster of differentiation 68; C1q, complement component 1q; ICU, intensive care unit; NA, not available; MAT, microscopic agglutination test.

**Table 2:** Clinical characteristics of AIN (Nussbaum *et al*., *Clin Kidney J* 2019) [49].

Fever	Rash	Eosinophilia (blood)	Triad of fever, rash and eosinophilia	Oliguria
Present in 15%–36% of cases	Present in 22%–27% of cases	Present in 23%–36% of patients	Present in up to 10% of cases	Present in 50% of cases

RBC, red blood cell.

**Table 3:** A comprehensive summary of the main publications pertaining to serum and urinary biomarkers of ATIN.

Reference	Population samples	Relevant findings	Association in distinguishing between different etiologies?
Dantas *et al*., *Kidney Blood Press Res* (2007) [52]	Glomerulopathy *n *= 37	The urinary MCP-1 was correlated with the extent of tubulointerstitial infiltrate by macrophages, but not with the degree of glomerular infiltrate	Until the present research, it was not sensitive, as it was found to be significantly higher in different etiologies [63, 92, 53, 59]
Wu *et al*., *Clin J Am Soc Nephrol* (2010) [53]	Drug-induced ATIN *n *= 40Healthy controls *n *= 20	ATIN patients had higher levels of MCP-1, α1-MG, NGAL and NAG compared with controls; the urinary levels of MCP-1were correlated with the extent and severity of the acute lesions	It is important to classify drugs based on their pathomechanism, since different drugs can induce ATIN through different pathomechanism which may aid in identifying the culprit drug
Nakashima *et al*., *Clin Nephrol* (2010) [54]	IgG4 disease-related ATIN *n *= 4Other cause ATIN *n *= 16	The expression of IL-4, IL-10 and TGF-β RNA in kidney tissue was observed to be higher in patients with IgG4 disease, as compared with other causes of ATIN	Due to the small sample size and the presence of such biomarkers in other autoimmune diseases, these are not universally applicable as a diagnostic biomarkers in IgG4-disease-related ATIN
Shi *et al*., *Am J Med Sci* (2013) [55]	Drug-induced ATIN *n *= 51	The rate of GFR decline was faster in patients with higher urinary levels of NAG, metalloproteinase 2 (MMP2) and MMP9	It is important to classify drugs based on their pathomechanism, since different drugs can induce ATIN through different pathomechanism which may aid in identifying the culprit drug
Aoyagi *et al*., *CEN Case Rep* (2014) [56]	One case of TINU	During follow-up of an episode of TINU, serum TNF-α, IL-8 and IFN-γ levels decreased	Several studies have found that these biomarkers are higher in various etiologies, making them non-sensitive and non-specific
Chen *et al*., *Braz J Med Biol Res* (2018) [57]	ATIN *n *= 30Healthy controls *n *= 15	The serum levels of IL-6, IL-10 and TNF-α were significantly higher in ATIN patients than in controls	Several studies have found that these biomarkers are higher in various etiologies, making them non-sensitive and non-specific
Zhao *et al*., *Am J Physiol Renal Physiol* (2019) [58]	ATIN *n *= 44Healthy controls *n *= 24	ATIN patients had higher urinary levels of KIM-1 and C5b9 compared with healthy controls; urinary C5b9 correlated with the extent of tubulointerstitial infiltrates in kidney biopsy in ATIN patients	Several studies have found that these biomarkers are higher in various etiologies, making them non-sensitive and non-specific
Yun *et al*., *BMC Nephrol* (2019) [59]	ATIN *n *= 113Healthy controls *n *= 40	Serum IL-1β, IFN-α2, TNF-α, MCP-1, IL-8, IL-17A, IL-18 and IL-23 were higher in ATIN patients compared with healthy controls; urinary IFN-α2, MCP-1, IL-6, IL-8, IL-12p70 and IL-17A were higher in ATIN patients compared with healthy controls	There are various biomarkers that may be due to the combined study of various etiologies; therefore, it is necessary to study among various etiologies in order to find specific biomarkers for each etiology
Moledina *et al*., *JCI Insight* (2019) [60]	ATIN *n *= 32Other kidney diseases *n *= 186	Urinary TNF-α and IL-9 were higher in ATIN patients compared with other kidney diseases; urinary IL-5 was higher among ATIN patients with prominent eosinophil infiltrates	ATIN vs other kidney diseases, cannot analyze such findings among various ATIN etiologies from this study
Moledina *et al*., *Nephron* (2019) [61]	ATIN *n *= 32ATN *n *= 41	Urinary TNF-α and IL-9 were higher in ATIN patients	ATIN vs ATN, cannot analyze such findings among various ATIN etiologies from this study
Moledina *et al*., *JCI* (2023) [62]	AIN *n* = 31Healthy controls *n* = 57	CXCL9 was significantly higher in AIN patients compared with healthy controls	It is therefore necessary to further study to determine the correlation between various etiologies of AIN

NAG: N-acetyl-neuraminidase; α1-MG: α1-microglobulin; NGAL: neutrophil gelatinase-associated lipocalin; TGF-β: transforming growth factor-β; GFR: glomerular filtration rate; KIM-1: kidney injury molecule.

## FEATURES OF INFECTION IN CORRELATION WITH AIN

Infection-induced AIN usually results in a sterile infiltrate, indicating that immunological disturbances may be the cause of AIN [65]. Therefore, in most instances, blood or urine culture may not be effective in identifying infection as an etiology of AIN (Table 1) [32–48]. An early diagnosis of the cause is essential to eliminate and prevent the responsible causes from occurring in the future [65]. Sonoda *et al*. [66] also found that local infection may induce an autoimmune response against autoantigens in the infected kidney, which could trigger organ-specific autoimmune disease. Therefore, it leads to confusion in determining whether infection is the root cause or autoimmune disease. Previously, infections were the primary causes of AIN; however, in recent times, an immuno-allergic mechanism triggered by medications such as antibiotics, NSAIDs and numerous others has emerged as the predominant cause [1].

Since infections and antibiotics induce AIN through autoimmune and immuno-allergic pathomechanisms, respectively, it is possible that their histopathological and clinical characteristics may overlap. According to some studies, the leading cause of AIN is bacterial pyelonephritis, which can be unilateral and typically localized to the renal pyramid [67]. In this instance, a healthcare professional can utilize positive uroculture, or, in most instances, blood cultures as a diagnostic tool for infection-associated AKI, particularly when AKl occurs in the absence of septic shock [67]. The presence of a large number of neutrophils [1], particularly as micro-abscesses, should alert one to the possibility of pyelonephritis [68]. The study conducted by Raghavan *et al*. [6] revealed a significant amount of neutrophilic infiltration, which tends to be negative on immunofluorescence microscopy. Such cases can present with both oliguric and non-oliguric renal insufficiency, without the classical clinical triad of AIN (fever, rash and arthralgia), usually with reversible renal function if infection is treated in a timely manner [37] (Tables 2 and 4 [49]). In patients with infection-induced AIN, steroids may increase the risk of immunosuppression, which could worsen the infection. Therefore, steroids should be delayed until the active infection is completely controlled [13–15] (Table 1 [38–48]).

**Table 4:** Urinalysis in AIN (Nussbaum *et al*., *Clin Kidney J* 2019) [49].

Basic urinalysis	Urine microscopy	Urine eosinophils	Urine chemistries
Proteinuria: present in 90% of cases	WBC casts: present in 3%–14% of cases	Sensitivity: 31%	FENa: can be >1% or <1%
Nephrotic-range proteinuria rare	RBC casts: present in up to 29% cases	Specificity: 68%	FEUrea: can be >35% or <35%
Hematuria: present in 50% of cases	RTE and granular casts: present in up to 86% cases	Also present in ATN, GN and other renal diseases	
Pyuria: present in 50%–80% of cases	Bland urine sediment: present in 20% cases		

RTE: renal tubular epithelial; GN: glomerulonephritis; FENa: fractional excretion of Na; FEUrea: fractional excretion of urea.

## FEATURES OF ANTIBIOTICS ASSOCIATED WITH AIN

The capacity to elicit an immune response to β-lactams (BLs) can exhibit varying degrees of variability: some patients exhibit a specific response to a specific BL and demonstrate tolerance to others, whereas other patients exhibit a universal response to all BLs [69] (Table 1 [32–38]). This phenomenon may be attributable to the fact that distinct patients may possess distinct HLA alleles that exhibit distinct responses to these BLs. Therefore, further investigation is necessary to identify specific HLA alleles that are more susceptible to AIN [64]. Perazella *et al*. [1] observed relatively short duration of exposure to the causative BL antibiotic, ranging from a few days to a few weeks, with the classical clinical triad of fever, rash or eosinophilia in >75% of patients, and the occurrence of proteinuria, leukocyturia or hematuria in approximately 75% of affected patients. AIN can also occur in association with the administration of non-BL antibiotics, such as rifampicin, when administered intermittently to treat mycobacterial infections (Table 1 [32–48]). The occurrence of AIN caused by sulfonamide antibiotics may be associated with typical hypersensitivity reactions, including fever, rash and eosinophilia [70]. Patients who have been infected with HIV, transplant recipients and patients who have pre-existing renal disease are at a higher risk of developing sulfonamide-induced AIN [71, 72]. The fluoroquinolone antibiotics, especially ciprofloxacin, can induce AIN without hypersensitivity syndrome [1].

Renal pathologists suspect the drug as an offending cause when a significant eosinophilic infiltrate (>10 eosinophils per 20× field) is present [1]. Immunofluorescence microscopy is typically negative in patients with AIN, although a very few cases of methicillin-induced AIN with tubular basement membrane deposits of immunoglobulin have been reported [73]. Immune complex deposits are relatively uncommon in drug-induced AIN—they have been reported with drugs such as methicillin and rifampin [73–75], which exhibit linear or granular staining for immunoglobulin G and C3 on tubular basement membrane (anti–tubular basement membrane antibodies). For example, the presence of hypersensitivity may be due to drugs such as BLs or sulfonamides. Asim *et al*. [76] presented a case of hemorrhagic tubulointerstitial nephritis caused by amoxicillin–clavulanate, characterized by systemic eosinophilia, a high concentration of eosinophils and plasma cells in the interstitial infiltrate, indicating a delayed hypersensitivity immune response mediated by antigen-reactive T cells. This case demonstrates an idiosyncratic immune response, with no correlation between the dose or the duration of amoxicillin–clavulanate therapy [6, 14, 77]. Although the alternative medication is typically recommended in cases of medication hypersensitivity associated AIN [3], it is important to note that there may be cross-reactivity between the two drugs. For instance, a case of drug reaction with eosinophilia and systemic symptoms (DRESS) syndrome was reported, wherein cross-reactivity was observed between vancomycin and subsequent teicoplanin administration [78], thereby enhancing the difficulty in determining the precise etiology. Similarly, several studies have found that patients with penicillin allergy have a higher risk of developing cephalosporin hypersensitivity associated AIN, but this finding is not universal [25, 79, 80]. The structural features of BLs could be the reason for this, as all BLs contain a five- or six-member ring, with the exception of monobactams (e.g. aztreonam), which have no reported cross-reactivity with penicillins [20].

## CLINICAL HISTORY AND PHYSICAL EXAMINATION IN RELATION TO INFECTION AND ANTIBIOTICS

A careful history can provide a clue about the offending agent but not a definitive confirmation, as previously described (Tables 1, 2 and 4 [32–48, 49]). For instance, the onset of either a mild or intermittently spiking fever typically occurs within a span of 2 weeks following the administration of a medication [1]. However, in the event that the patient has been previously exposed to the aforementioned drug, the duration of fever may be limited to a few hours or days [25]. The occurrence of fever as a systemic feature of AIN is variable, and the rate of variation is contingent upon the class of drug involved [1] (Table 1 [32–38]). Fever may be absent in AIN caused by many drugs, such as NSAIDs and non-BL medications, but it has been manifested in as many as 50%–100% of patients with AIN caused by penicillin derivatives, particularly methicillin [25, 77, 80–82]. The occurrence of AIN-associated rash is observed in 15%–50% of patients with drug-induced AIN, particularly with agents that can trigger a hypersensitivity reaction, such as penicillin derivatives, sulfonamides, allopurinol and phenytoin [11, 16, 80]. Patients receiving rifampicin frequently experience symptoms of flank pain and tenderness, resulting from the distention of the renal capsule caused by inflammation and parenchymal swelling [83, 84].

## CLASSICAL BIOMARKERS OF AIN IN RELATION TO INFECTION AND ANTIBIOTIC

### Commonly ordered tests

A series of urine and serum tests is frequently ordered to assess and distinguish the precise cause of AIN. According to a recently published study, dipstick pyuria was observed in 60–80% of AIN cases caused by antibiotics [10] (Tables 2 and 4 [49]). However, the absence of pyuria does not exclude the diagnosis [63]. Numerous studies on AIN have reported the presence of mild to moderate proteinuria, which is incapable of distinguishing between infection or antibiotic as the causative agent, or among other AIN etiologies [49]. However, nephrotic-range proteinuria is rare [4, 5, 10, 28, 85, 86], and when found in the setting of AIN, it should raise suspicion for NSAID-induced nephrotoxicity or underlying glomerular diseases [5, 10, 87, 88]. Hematuria is also a nonspecific and insensitive laboratory test, despite several studies indicating a mean of 50% (with a range of 20%–80%) in cases of AIN [10, 85]. It is more common with some drugs, especially methicillin and the BL class of antibiotics, which can cause hematuria in up to 90% of patients with BL antibiotic-induced AIN [25]. The presence of white blood cell (WBC) casts has been demonstrated in the context of intrarenal infection, such as pyelonephritis, or in conjunction with inflammatory renal lesions, such as proliferative glomerulonephritis and AIN, rendering them non-specific and insensitive for AIN [49]. Numerous studies have revealed that eosinophilia was observed in 80% of cases of drug-induced AIN caused by BL antibiotics, such as methicillin, while it was only observed in less than one-third of cases of drug-induced AIN caused by non-BL antibiotics [16, 80, 89]. Eosinophiluria cannot be used in diagnosing AIN because it is detected in many renal and nonrenal diseases such as pyelonephritis, cystitis, prostatitis, atheroembolic disease, acute tubular nephritis, rapidly progressive glomerulonephritis and bladder malignancies, among others [50, 73, 90, 91].

### Novel biomarkers

In recent times, a variety of novel biomarkers have been evaluated for the identification of the distinct characteristics of interstitial infiltrates and the cytokines produced by this infiltrate, in relation to other inflammatory renal diseases (Table 3). Their detection in serum or urine has been identified as potential diagnostic and prognostic tools for ATIN. The presence of specific chemokines and cytokines can provide a clue towards a potential causative agent, as the pathophysiologic process may vary depending on the etiology. For instance, Moledina *et al*. [62] observed a higher level of the major eosinophilic attractant C-C motif ligand 3 (CCL3) in patients with AIN compared with the control group. This finding may serve as a means to distinguish patients with AIN due to antibiotics, as they are known to have a higher number of interstitial eosinophils. On the contrary, it is possible that the level of monocyte chemoattractant protein-1 (MCP-1) is elevated in infection-induced AIN due to its significant role in the recruitment of monocytes, neutrophils and lymphocytes in tissue inflammation processes [63]. Furthermore, several studies have reported a higher level of neutrophils in infection-induced AIN [6]. However, it is not feasible to assess its sensitiveness or specificity as it has been identified as a chemokine implicated in numerous autoimmune diseases [92]. It is noteworthy that Muhammad *et al*. [93] hypothesized that drugs may be able to induce autoimmune diseases that induce AIN. Therefore, researchers are uncertain about the causative agent that triggers the MCP-1 level in such a scenario. Wu *et al*. [53] also observed a higher level of MCP-1 in a cohort of 40 patients with drug-induced AIN in comparison with controls. Similarly, Yun *et al*. [59] found a higher level of MCP-1 in serum and urine in 113 patients with AIN, resulting from different causes, through a bead-based multiplex assay. Despite the findings of Moledina *et al*. [60, 62, 94], who observed a higher level of urinary C-X-C motif ligand 9 (CXCL9), interleukin-9 (IL-9) and tumor necrosis factor-α (TNF-α) levels in patients with AIN, these biomarkers are unable to definitively identify the causative agent among various etiologies. As IL-9 and IL-5 are implicated in allergic responses and promotes mast cell accumulation, which in turn is a source of TNF-α [60, 61, 95–98], it is plausible that these cytokines, along with CCL3, may provide a clue in identifying the culprit drug in hypersensitivity-associated AIN. In a similar context, elevated levels of IL-6 and IL-8 may indicate infection as the causative agent of AIN [59, 98–101]. However, there are no published studies that specifically demonstrate the significance of these cytokines as indicators of the offending agent.

### Cellular biomarkers

The binding of T cells to a drug is a complicated process that can be measured through cellular assays based on the demonstration of the lymphocyte proliferation response and the cytokine secretion response when exposed to the suspected drug [63]. Fluorescence cytometry techniques can be employed to assess the activation phenotype of lymphocytes subsequent to drug exposure. Several studies have demonstrated the detection of activation markers on the surface of lymphocytes subsequent to incubation with the offending drug, such as CD25, CD69 or HLA drug reaction (DR), indicating hypersensitivity [95, 101].

A sensitive test, the enzyme-linked immunospot (ELISpot) assay, has been reported to be useful in evaluating drug hypersensitivity reactions. It allows the *ex vivo* measurement of the release of cytokines by lymphocytes in response to a certain stimulus [102]. There is, however, limited evidence regarding the usefulness of this assays specifically in patients with AIN. Punrin *et al*. [103] reported that 50% of patients with drug-induced ATIN had a positive interferon (IFN)-γ ELISpot assay in a cohort study.

### Lymphocyte transformation test 

It is possible to use the lymphocyte transformation test (LTT) to measure the proliferation of lymphocytes in response to the pure form of a suspicious drug. Koda *et al*. [104] reported the utilization of LTT in identifying the culprit drug in ATIN among lansoprazole and nivolumab, and it demonstrated reactivity against lansoprazole, but not against nivolumab. Positive LTT has also been reported in the context of ATIN induced by BLs and NSAIDs [98]. Nonetheless, the LTT concept can yield accurate results only if drugs are able to directly interact with the T-cell receptor and act as hapten, without any prior metabolism or binding to proteins [98]. Beta-lactams are known to be able to act as haptens and bind to amino groups of amino acids, such as lysine [105]. However, clinicians should understand that sometimes the reaction is not caused by the drug itself, but by a component within the drug or a metabolite which transforms this so-called pro-hapten to hapten [106], which may result in negative LTT. Sulfamethoxazole is a typical example of a drug that acts as prohapten, because it is transformed to sulfamethoxazole–hydroxylamine and further oxidized to sulfamethoxazole–nitroso [107–109]. Drugs that exhibit pseudoallergic responses in nonimmune-mediated hypersensitivity reactions by affecting the innate immune system and/or effector cells, such as basophils directly, may also be tested negative, without any evidence of involvement of the specific immune system [98]. The possible drawback of this method could be the long time it takes for the procedure to display the result, because in the acute setting, the LTT has a low specificity because sensitization does not always associate disease [110]. Chung *et al*. [111] confirmed through LTT that vancomycin, but not other glycopeptide antibiotics, specifically induced T-cell proliferation in the reported case.

### Genetic biomarkers

Some studies have described the association of AIN, mainly in the setting of TINU, with certain HLA or single-nucleotide polymorphisms as markers of disease susceptibility. According to a study conducted in a cohort of 154 Chinese patients with different causes of AIN, HLA-DQA1*0104/DQB1*0503/DRB1*1405 are risk haplotypes for the development of AIN [64]. These studies suggest that these variants may enhance antigen presentation and facilitate renal interstitial inflammation. Therefore, it is important to determine which specific HLA alleles are more susceptible to which etiology of AIN. We may consider combining this HLA testing for diverse etiologies of AIN with adjunctive testing, such as IFN-γ ELISpot, to obtain a more favorable outcome.

## DISCUSSION

This narrative review demonstrates the relevant features of ATIN in the setting of infection, as opposed to the situation of a drug-induced immune allergic reaction, with a special focus on the antibiotic-associated ATIN. The main purpose of the study was to analyze the peculiar findings that are typical of an infection-associated ATIN compared with an antibiotic-associated ATIN caused by an allergic reaction. Although these findings cannot provide conclusive and clear recommendations that can be useful in the clinical practice, they might encourage researchers to conduct original research on these features to find clear recommendations.

Hospital-acquired AKI affects up to 10%–27% of patients affected by AIN, which has various etiologies, among which antibiotics and infections account for up to 35% and 16% of cases, respectively [4–9]. Both etiologies necessitate distinct therapeutic measures in a timely manner, as previously described [13–15]. The delay in identifying the precise culprit agent can impede therapeutic measures, ultimately leading to renal fibrosis and CKD [1, 3, 11, 13, 18, 19]. Previous studies have demonstrated that, in up to 30% of cases, the culprit drug cannot be precisely identified, and this type of misdiagnosis can lead to partial renal recovery, recurrent disease or CKD in up to 40%–60% of cases [20–25]. Therefore, a timely and thorough investigation is required for complete recovery. Currently, there is no universal procedure that can be utilized to determine the precise cause, despite the fact that certain clinical and histopathological features may provide a clue towards the potential suspected cause, as previously described. Therefore, it is imperative for clinicians to exercise caution in selecting the appropriate therapeutic measures, particularly when both infections and antibiotic-related causes are suspected; however, they treat patients on the basis of clinical history, physical examinations and biopsy findings, without caring much about the exact etiology.

We have highlighted various characteristics associated with antibiotics and infections that give clues, but cannot be deemed universal findings. For example, an increase in neutrophil levels points towards infection, while an increase in eosinophils points towards hypersensitivity to drugs [6]. In contrast, an increase in neutrophils can also be caused by urinary tract infections. Similarly, different antibiotics exhibit different hypersensitive reactions in different patients, with some individuals exhibiting tolerability to certain drugs while others exhibit hypersensitivity to other drugs. This can be challenging to discern when taking multiple antibiotics. Clinicians also follow the typical triad of fever, rash and arthralgia, but it is not always helpful. The time period from drug exposure, which typically ranges from 7–10 days, is utilized as a diagnostic indicator [20]. However, this method is not universally accurate for all drugs, particularly when a patient is exposed to the same drug for the second time, where the hypersensitivity reaction can be more rapid, as previously described. In cases of suspected infection, some clinicians also rely on blood or urine culture, but this may not be effective because it usually results in a sterile infiltrate, as infection induces AIN primarily through immunological disturbances. Thus, both etiologies may have similar histopathological and clinical characteristics due to the same immunological pathomechanism. Clinicians may also prescribe alternative medications in case of hypersensitivity to one drug, but there may be cross-reactivity between the previous drug and the alternative, which may be misleading in finding the offending agent.

Various serum and urine tests, including dipstick pyuria, proteinuria, hematuria and WBCs, are routinely ordered and relied upon to distinguish between the two etiologies, however they are neither sensitive nor specific, as previously described. Numerous novel biomarkers including CCL3, CXCL9, MCP-1, TNF-α, IL-9, IL-5, IL-6 and IL-8 have been evaluated for the identification of the distinct characteristics of interstitial infiltrates in different etiologies. However, they are not routinely performed and are not recommended at universal guidelines. There are some reports that indicate the utility of the ELISpot assay in evaluating drug hypersensitivity reactions [64]. However, there is limited evidence regarding its efficacy in patients with AIN. The LTT possesses several drawbacks, including the inability to display results promptly and its inability to be utilized across all classes of drugs due to the variability of drug metabolism, rendering it unsuitable for routine use. Several studies have published associations between ATIN and certain HLA or single-nucleotide polymorphisms as markers of disease susceptibility, mainly in the setting of TINU [64]. However, there is currently no further investigation into the identification of HLA risk alleles in other causes of AIN, which is crucial for determining the prognosis of AIN.

In brief, our study does not provide conclusive and clear recommendations that can be useful in the clinical practice, and further research is required regarding the features described above.

## Data Availability

All data included in this study are available upon request from the corresponding author.
